# Evaluation of Electrospun Nanofiber-Anchored Silicone for the Degenerative Intervertebral Disc

**DOI:** 10.1155/2017/5283846

**Published:** 2017-10-18

**Authors:** M. Khandaker, S. Riahanizad

**Affiliations:** Department of Engineering and Physics, University of Central Oklahoma, Edmond, OK 73034, USA

## Abstract

The nucleus pulposus (NP) substitution by polymeric gel is one of the promising techniques for the repair of the degenerative intervertebral disc (IVD). Silicone gel is one of the potential candidates for a NP replacement material. Electrospun fiber anchorage to silicone disc, referred as ENAS disc, may not only improve the biomechanical performances of the gel but it can also improve restoration capability of the gel, which is unknown. This study successfully produced a novel process to anchor any size and shape of NP gel with electrospun fiber mesh. Viscoelastic properties of silicone and ENAS disc were measured using standard experimental techniques and compared with the native tissue properties. Ex vivo mechanical tests were conducted on ENAS disc-implanted rabbit tails to the compare the mechanical stability between intact and ENAS implanted spines. This study found that viscoelastic properties of ENAS disc are higher than silicone disc and comparable to the viscoelastic properties of human NP. The ex vivo studies found that the ENAS disc restore the mechanical functionality of rabbit tail spine, after discectomy of native NP and replacing the NP by ENAS disc. Therefore, the PCL ENF mesh anchoring technique to a NP implant can have clinical potential.

## 1. Introduction

Current surgical treatments such as discectomy, spinal fusion, and total disc arthroplasty are complex and costly, failed to restore normal range of motion, and failed to permanently relieve chronic lower back pain [1–3]. Emerging techniques as an alternative to the latter include NP replacement by polymeric hydrogels. This aims at replacing only the degenerated NP, keeping the remaining disc structure intact [4]. NP replacement by tissue-engineered grafts is still developing, since cell and molecular biologists are still struggling to determine the nature of the NP cells [5]. The NP substitution by polymeric hydrogel is one of the promising techniques for the repair of degenerated IVD [6]. Polymeric hydrogels, such as silicones [7], polyurethanes [8], polyethylene [9], polyvinyl alcohol [10], and polymers reinforced with fibers or ceramics [11], have been investigated as a suitable replacement material for NP. Though these polymers exhibit similar characteristics like the natural IVD in terms of biomechanics behavior, inadequate stiffness and cellular responses between the implant and biological environment make them inappropriate materials for IVD restoration [12]. Despite the intensive research over the past decade about polymeric IVD fusion materials and joint soft tissue regeneration, there is not yet any material that can reproduce adequately the physiological, mechanical, and biological behavior of the natural NP, at the same time exhibiting long-term functionality when introduced into the human spine [6]. Development of a tissue-engineered intervertebral disc, where nucleus pulposus implant will be anchored to annulus fibrosus and the end plate by tissue-engineered matrix, may have advantages over techniques.

A functional disc replacement material and implantation technique must restore the biomechanical functions of the disc after implantation [13]. Our previous study showed that silicone NP implants that are able to mimic the mechanical behavior of the native nucleus may still fail in restoring the disc stability after discectomy [14]. In order to obtain a biomechanical equivalent natural IVD, it requires proper anchoring of the NP implants and its replacement inside the disc [15]. Such anchorage may restore the disc stability after discectomy. The proper anchorage of a NP gel is possible by enclosing the NP by electrospun fiber mesh that mimics the surrounding structures. The appropriate design of the interface between the NP and its surrounding structures is also important for the migration of endogenous cells between the NP implant and native tissue to innate the repair of intervertebral discs after the IVD implantation [16]. Current state-of-the-art total IVD implant consists of NP implant enclosed by electrospun fiber that mimics the hierarchical organization of only native AF [17]. The biomechanical performance of such IVD implant can further be improved if electrospun fiber mesh can secure the NP implant in all sides. In addition, those fiber meshes act as scaffolds for both AF and EP cell growth after the in vivo implantation of ENAS in the spine. However, it is unknown whether ENAS surrounded by PCL nanofiber matrix can better restore the biomechanical functions of disc in comparison to intact discectomy (without NP condition) and those IVD made with silicone only. The above facts formulate the research design and methods for the objectives of this study. The successful achievement of the study will lead to the design of functional disc replacements.

## 2. Materials and Methods

### 2.1. Materials

Electrospun poly(e-caprolactone) (PCL) was used in the study for the anchorage of the silicone gel. The reason of using PCL fiber is that electrospun PCL seeded with either AF cells or mesenchymal stem cells has been proven to increase the functional properties with *in vitro* culture, approaching native IVD tissue properties at the single and multilamellar length scales [18, 19]. It is a suitable scaffold for tissue generation too.

The materials used for the silicone synthesis include responsive silicone gel system base (implant grade) and the responsive silicone gel system crosslinker (implant grade). The silicone gel system was selected to model the nucleus pulposus of the intervertebral disc. Silicone gel was prepared by mixing a 40 wt% of poly-dimethyl-hydrogen-siloxane crosslinker agent with polydimethylvinylsiloxane base. Both base and crosslinker were purchased from Applied Silicone Corporation. Silicone was used as the NP gels, since the previous investigation found that silicone gel has adequate viscoelasticity properties (compressive, viscoelastic properties) to be suitable for NP implant materials for IVD application. The rabbit spine model has been used for the *in vivo* feasibility study of IVD for disc restoration by other researchers.

### 2.2. Specimen Design

**Figure 1:**
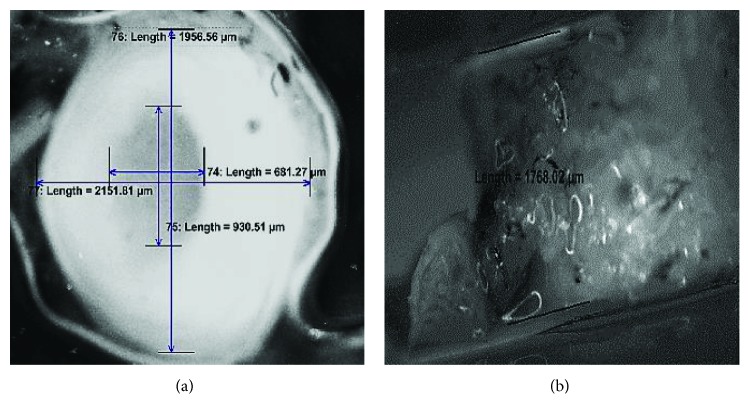
(a) Transverse and (b) longitudinal section images of a rabbit Cd4 vertebra that is used to measure the dimension of NP.

This study prepared two different kinds of discs that can be used as a replacement for degenerated NP. They are referred as silicone and PCL electrospun nanofiber-anchored silicone (ENAS) disc. The dimension of the disc was calculated from the size of the NP by dissecting a rabbit tail at caudal disc level 4 transversely and longitudinally with respect to the long axis of the tail (Figure 1). Compression and rheological tests were conducted on each group of discs. Also, ex vivo study was conducted on each group of samples for the restoration capability of the ENAS disc-implanted tail with respect to native tail.

### 2.3. Specimen Preparation

**Figure 2:**
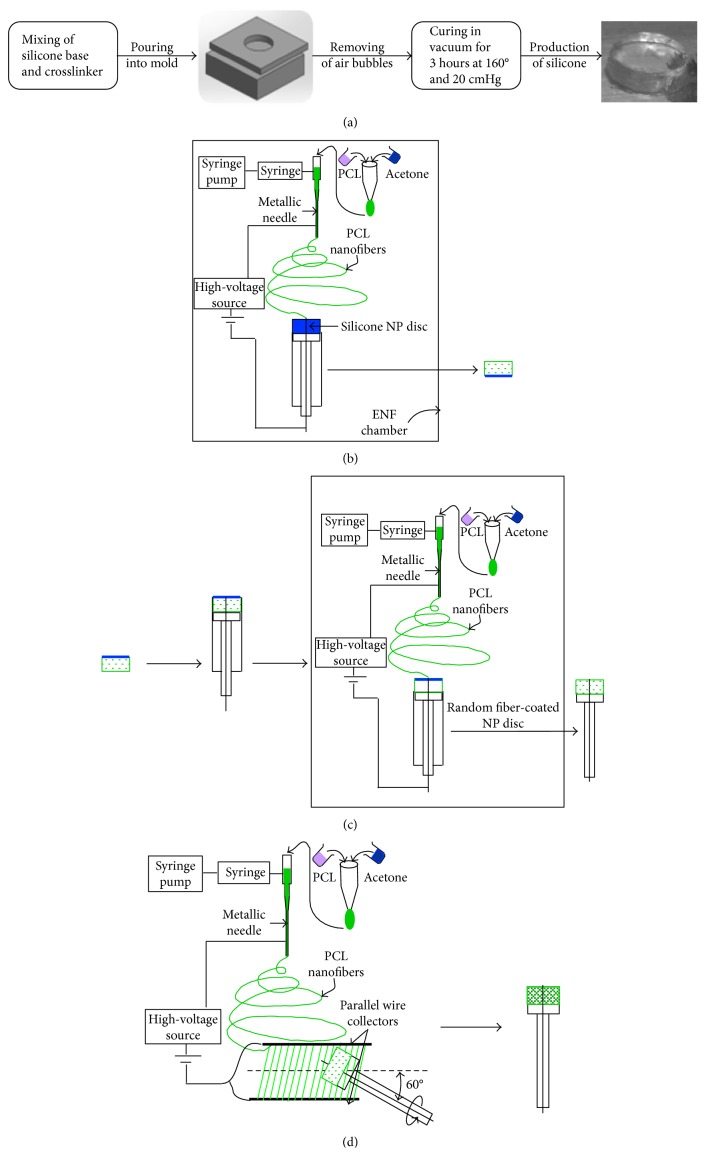
Process for the fabrication of ENAS disc: (a) fabrication of silicone disc, (b) coating of NP disc in top and circumferential sides by random fibers, (c) coating of NP disc by random fibers on the side where there is no existence of fiber coating, and (d) production of angle-ply structure on the circumference of NP disc.

The silicone disc preparation process is shown in Figure 2(a). The silicone was prepared using a 73.2% to 26.8% by weight ratio of silicone system base and silicone system crosslinker, respectively. The silicone base and silicone crosslinker were weighed and measured with the scale and VWR pipette, then mixed in 100 mL beaker for 20 minutes where the solution was stirred manually with a glass stirring rod. The solution was then placed in the VWR vacuum oven for 10 minutes at 20 cmHg vacuum to remove the air bubbles from the solution. Once the air bubbles were removed, the silicone solution was removed from the oven and into the aluminum molds with the use of the VWR pipette. The aluminum mold was then placed in the VWR vacuum oven at 160°C and 20 cmHg for 3 hours for curing. The silicone mold was allowed to cool and removed from the oven at which the silicone disc model was removed from the mold and prepared for nanofiber application. This study successfully fabricated 10 mm diameter and 5 mm thickness silicone gel using the process.

For the production of ENAS disc, silicone disc was prepared according to the previous method. The silicone disc was anchored by PCL ENF matrix by the following three-step process.

#### 2.3.1. Step 1: Coating on Top and Circumferential Surface of the NP Disc by Random Fiber

Silicone NP disc was coated with random fiber at the top and circumferential sides by multilayers of random fiber in the custom-made plastic syringe.  Figure 2(b) shows the coating of NP disc on top and circumferential sides by random PCL fibers. The embodiment shown in the diagram includes the sealed chamber, a syringe pump, a syringe with a tube that is attached with a nonconducting support, a syringe needle at the end of the tube, a high-voltage power supply, and custom-made plastic syringe containing the NP disc. The syringe needle is electrically charged by applying a high voltage in the range of 5 KVA to 15 KVA produced by the power supply. An opposed charge is applied to the conductive wire at the center of the custom-made syringe by applying a high voltage in the range generated by the power supply.

#### 2.3.2. Step 2: Coating on the Bottom Surface of the NP Disc by Random Fiber

The NP disc is placed again in the plastic chamber with the conductive wire inserted at the center. The bottom side of NP that did not have PCL fiber mesh is faced up so that the random fiber can be coated on that side. Multiple layers of random fibers are deposited on the side.  Figure 2(c) shows the coating of NP disc by random fibers on the side where there is no existence of fiber coating. The resultant product from the step was a NP disc where every side was anchored by multilayers of random fiber.

#### 2.3.3. Step 3: Coating on Circumferential Surface of the Disc by Aligned Fiber

The process concludes with the disc being completely encased with an electrospun nanofiber membrane to enhance the osseointegration of NP disc. The disc is wrapped with a 60-degree angle-ply band constructed of electrospun microfibers to mimic natural IVD annual fibrosus (AF) (Figure 2(d)). The AF that comprises discrete fibrous sheets with specialized collagen alignment bears the multidirectional loads around the circumference. Fibers run in a single direction in native AF tissue, ranging from 20° to 60° with respect to the transverse plane, and adjacent lamellae have opposing fiber orientations, producing an angle-ply structure.

The angle-ply fibrous structures were produced along the circumference of the NP disc by rotating the plastic syringe plunger that contains the disc. Aligned PCL angle-ply structure was deposited on the NP disc by manually rotating the NP disc 6 times (+ and −60 degrees).

### 2.4. Mechanical Characterization

Evex compression test setup was used to find the silicone gel and ENAS disc compressive modulus. Both samples were compressed to 80% of the gel height at a rate 0.05 mm/sec during the unconfined compression tests. Oscillation tests were performed on the hydrogel using the Malvern CVO-100 rheometer at 5% strain rate at frequency 1 Hz. Viscous, elastic, and complex modulus for each group of discs were found from the experiment. Phase angles were also determined from the experiments.

### 2.5. Ex Vivo Animal Tests

**Figure 3:**
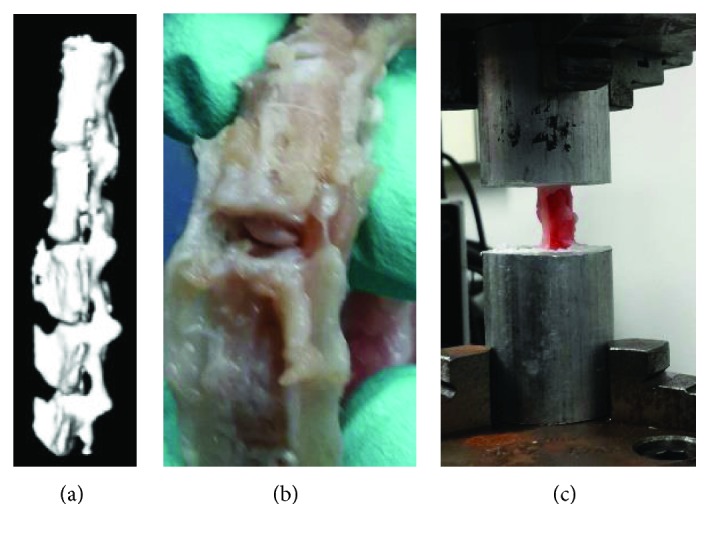
(a) CT scan image of a rabbit tail, (b) ENAS disc implantation on rabbit tail, and (c) sample in a test resource UTM.

Caudal (Cd) vertebral (Cd1–Cd7) spines were collected from female New Zealand white rabbits. All rabbits were euthanased when approximately 3 months old (weight range, 3.2–3.8 kg) as part of the authors' ongoing grant support from OK-INBRE (Oklahoma IDeA Network of Biomedical Research Excellence). The experiment has been approved by the Institutional Animal Care and Use Committee (IACUC), University of Oklahoma Health Sciences Center. Each rabbit had a titanium implant on the femur for 8 weeks, and there was no known history of back pathology. CT scan images (Figure 3(a)) of spinal specimens were used to identify the location of discectomy and sample insertion site at the caudal vertebral column. Immediately after euthanasia, the spinal specimens were harvested and frozen at −20°C. Prior to testing, each frozen specimen was thawed at 4°C (24 h) and cleared of all soft tissue. The specimens were divided into two groups, with group 1 containing a native spinal specimen and group 2 containing spinal specimen after discectomy and insertion of the ENAS disc at Cd4. The annular incision was closed using Vetbond Tissue Adhesive (3M™ Animal Care).

The Cd2 vertebra was embedded in a metal cup by BioMedtrix 3 veterinary bone cement. After 30 minutes of curing of the cement, the embedded spinal specimens were fastened at the top gripper in Test Resources UTM. Another same size metal cup was secured in the bottom gripper, and bone cement was poured into the cup. The top metal cup with the embedded spine was lowered till the Cd5 is completely covered by the bottom cup cement. Due to low viscosity of the bone cement, negligible preload was applied to the spine during the anchoring of the spine to the bottom cup (Figure 3(c)). After 30 minutes of curing, each sample was compressed by 5 sequential 3% of strain increments from 3% to 15% strain. After each increment, the load and displacement data were recorded.

## 3. Results and Discussion

**Figure 4:**
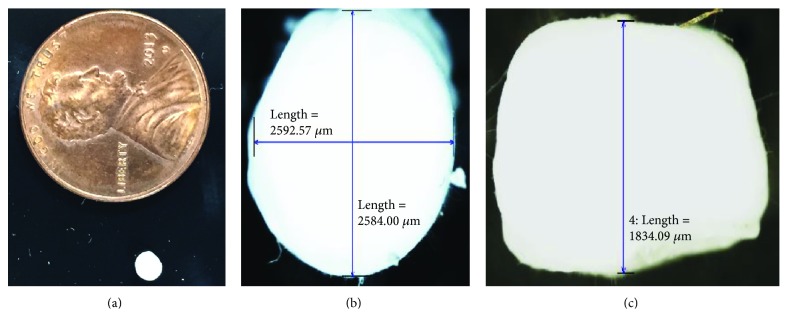
(a) Fabricated ENAS disc, (b) circumferential dimensions of the fabricated disc, and (c) height of the disc.

This study successfully produced an ENAS disc (Figure 4(a)). Figures 4(b) and 4(c) show the dimension of the fabricated disc. The size of the disc closely matches the size of NP in the spine of a rabbit tail (Figure 1). Any size of IVD can be produced using the unique electrospun technique.

**Figure 5:**
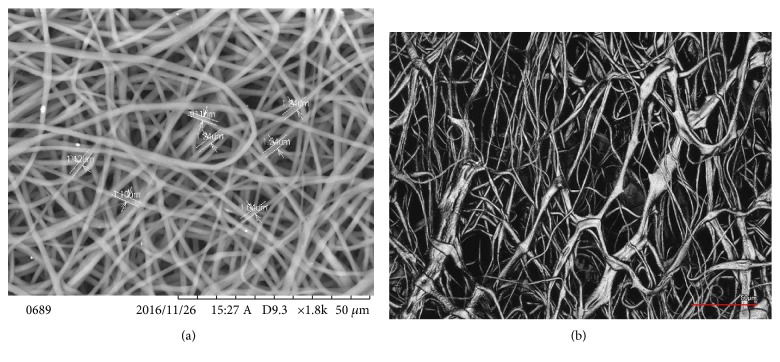
Distribution of PCL ENF matrix on silicone in ENAS disc: (a) top/bottom sides and (b) circumferential side.

Figure 5(a) shows the SEM images of the deposited PCL ENF matrix at the top/bottom sides of the ENAS disc.  Figure 5(b) shows the SEM images of the deposited PCL ENF matrix at the circumferential side of the ENAS disc. The diameters of the fibers in the matrix were found to be in the range from 0.9 *μ*m to 1.24 *μ*m.

**Figure 6:**
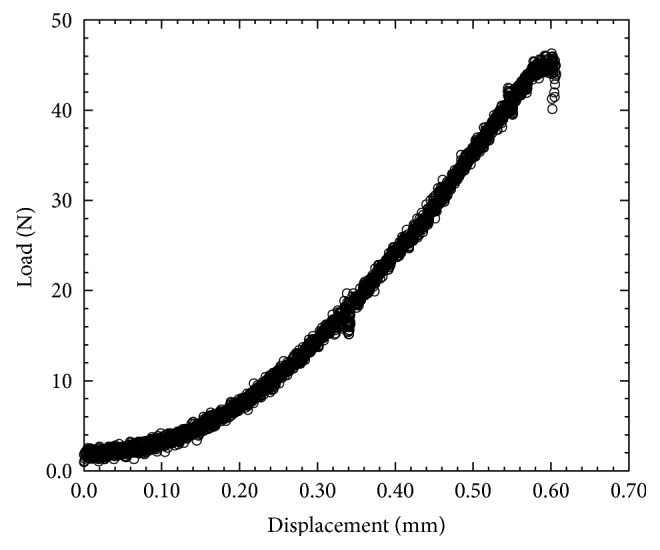
Load versus displacement curve of an ENAS disc from compression test.

**Figure 7:**
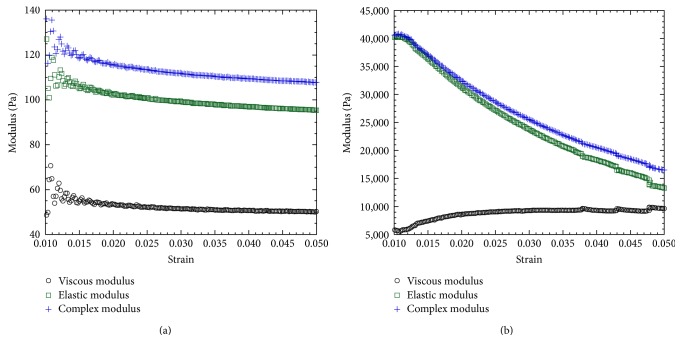
Rheological test results of a (a) silicone and (b) ENAS disc sample showing the higher values of modulus with respect to strain.

This study found that the compressive modulus, complex shear modulus, and phase shift angle of silicone gels and EIVD are in the range of human NP. These results confirm the suitability of electrospun fiber anchorage to gels, since it improves the mechanical properties of the gels. The compression test results show that ENAS disc compressive modulus (87.47 ± 7.56 kPa, *n* = 3) was significantly higher in comparison to silicone gel (38.75 ± 2.15 kPa, *n* = 3) and the value was within the range of the compressive modulus of human NP (64.9 ± 44.1 kPa [20]). The maximum compressive pressure of ENAS disc was measured from the load-displacement curve (Figure 6), which was 0.46 MPa. Since the range of resting compressive stress for rodent animal tails is 0.2 ± 0.05 MPa [21], therefore, our designed ENAS disc is adequate for the proposed study. The rheological test results (Figure 7) show that ENAS disc elastic modulus (16~40 kPa) is significantly higher in comparison to silicone gel (0.10~0.16 kPa) and the value is within the range of the compressive modulus of human NP (7~20 kPa [22]). These results confirm the suitability of ENAS disc over silicone as NP implant.

**Figure 8:**
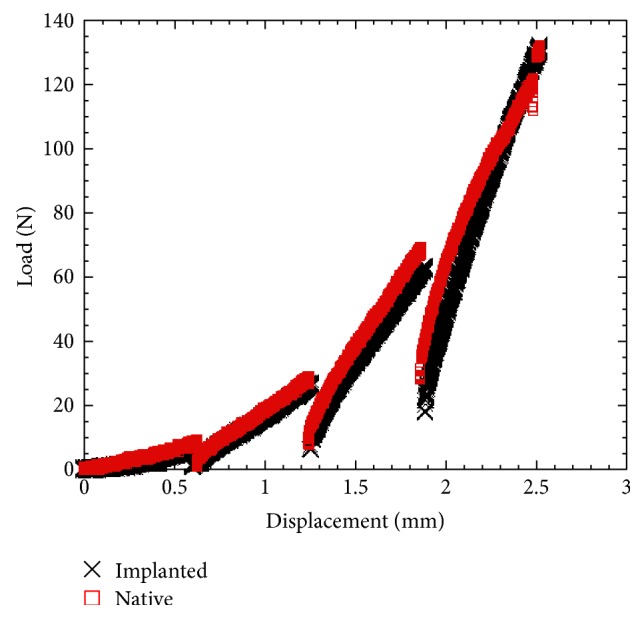
Load-displacement curve of native and implanted samples.

It is evident from comparing the load versus displacement curves of the spine without and with ENAS disc (Figure 8) that ENAS was able to restore the compressive load behavior of native rat caudal spine. There was no leakage of silicone observed during the compression of the spine with ENAS samples till 15% strain level. Further in vivo studies are required to evaluate the effect of osseointegration on the restoration capability of the normal biomechanics after the implantation of the ENAS disc in an animal model.

This study developed and evaluated a fabrication process of encapsulating the NP with an AF and endplate region produced via PCL ENF mesh. This study provided novel information on the function of PCL ENF mesh with the exclusive enclosure of silicone in every side. Studies provide a better understanding of how the PCL ENF anchorage selection on silicone NP influences mechanical behavior. Producing an engineered IVD capable of integrating with native structures robustly would advance the idea of tissue-engineered IVD to clinical application. This research has an opportunity for the incremental advancement in technology for the design of novel IVD for human and animal model. This study has multiple alternative strategies and can be adopted in case the proposed method does not overcome the technical hurdle of poor mechanical stability and osseointegration.

Development of a tissue-engineered IVD, where NP implant is anchored to the AF and end plate, is innovative and may have advantages over other existing techniques for the treatment of the degenerated IVD diseases. The fully enclosed NP within the engineered construct with alternative layers of nonbiodegradable (such as polystyrene and nylon 6) and osteoconductive (biomineralized PCL) materials may provide improved NP retention and integration with tissues. The effect of implantation time period and IVD structure (without and with biomineralized PCL) on the restoration of the biomechanical function of the spine after discectomy will be identified as a translation for this study. This study provided a unique material and structural information that will be used for the design of novel IVD implant for humans in the future.

## 4. Conclusion

This study successfully produced a novel method of anchoring a NP gel, in this study silicone, using electrospun fiber mimicking the size of NP at rabbit tail spine. Any size of engineered IVD can be produced using the unique electrospun nanofiber technique (patent pending [23]). Angle-ply fiber cloth mimicking the AF architecture is also produced using the method. The results suggested that ENAS disc is suitable as the replacement disc and should be studied further for the feasibility in IVD application. Dynamic mechanical behavior of the motion segments after in vivo implantation of ENAS disc will provide further proof of the restoration capability of the ENAS disc.
